# How Financial Challenges Affect Health and Lived Experiences of Adults With Long-Term Services and Supports Needs

**DOI:** 10.1093/geroni/igaf122.3956

**Published:** 2025-12-31

**Authors:** Lei Chen, Qianyun Wang, Kathryn Kietzman

**Affiliations:** University of California, San Francisco, San Francisco, California, United States; University of California, Los Angeles, Los Angeles, California, United States; UCLA Fielding School of Public Health, Los Angeles, California, United States

## Abstract

Older adults and people with disabilities living at home with chronic care needs may require Long-Term Services and Supports (LTSS). However, little is known about the diverse financial challenges they face due to these care needs, and how these challenges may impact their health and daily lives. This study draws on analyses of population-level quantitative survey data (2019-2020, n = 2030) and a subgroup of 18 in-depth qualitative interviews (2023-2025, n = 90) collected as part of the California Long-Term Services and Supports study. Employing a sequential mixed-method approach, we used descriptive statistics and grounded theory to identify financial challenges experienced by adults with LTSS needs and examine how these challenges affected their health and well-being. Survey findings show that 61% of California’s adults with LTSS needs experienced financial worries that were associated with their self-rated health and serious psychological distress. We also found an association between lower incomes and poorer health outcomes. Qualitative interview participants identified key financial challenges including: barriers to accessing public benefits (e.g., age, employment, or education restrictions); inadequacies in caregiving and LTSS services (e.g., insufficient caregiving hours); services, supports, and equipment not covered by insurance; high housing cost and poor housing quality; and heightened vulnerability to financial insecurity. These results highlight the urgent need to refine LTSS eligibility criteria and integrate appropriate age, housing, employment, and income considerations into policy development. Findings also underscore the importance of developing inclusive programs that reduce financial disparities and strengthen the quality of care provided to people with LTSS needs.

